# Factors contributing to anxiety in adolescents surviving thalassemia major in Indonesia

**DOI:** 10.1186/s12887-025-05403-3

**Published:** 2025-01-31

**Authors:** Henny Suzana Mediani, Novitasari Tsamrotul Fuadah

**Affiliations:** 1 Department of Pediatric Nursing, Faculty of Nursing, Universitas Padjadjaran, Sumedang, 45363 West Java Indonesia; 2 Faculty of Nursing, Bhakti Kencana University, Bandung, West Java Indonesia

**Keywords:** Adolescent, Anxiety, Psychological, Thalassemia Major

## Abstract

**Background:**

Thalassemia major is a significant public health concern, passed from parents to children, which can be mitigated through screening programs. Inconsistencies in blood transfusions and iron chelation therapy result in physical changes that can cause psychological problems, with anxiety being the most prominent. This study aimed to examine the factors influencing anxiety levels among adolescent thalassemia major survivors.

**Methods:**

The research utilized a quantitative approach with a correlational analytic design and cross-sectional method. It included a population of 122 adolescent survivors, all of whom were included using a total sampling technique. Data analysis involved univariate analysis by frequency distribution, bivariate analysis using the chi-square test, and multivariate analysis with logistic regression.

**Results:**

The findings of the study showed that adolescent thalassemia survivors experienced varying levels of anxiety: mild anxiety in 70.5%, moderate anxiety in 9.8%, and severe anxiety in 19.7%. Significant associations were observed between anxiety levels and factors such as body image (*p* < 0.001), self-esteem, and coping strategies, while social support did not show a significant relationship. Body image was identified as the most impactful factor, with poor body image raising the risk of severe anxiety by 11.6 times.

**Conclusions:**

Anxiety is common among adolescent thalassemia survivors, with body image, self-esteem, and coping skills being key factors. Poor body image notably increases the risk of severe anxiety, emphasizing the need for psychological support focused on body image, self-esteem, and coping strategies to improve mental health in this group.

## Introduction

Thalassemia major typically presents with fetal hemoglobin (HbF) levels ranging from 75 to 98% and hemoglobin A (HbA) at around 2%. In this condition, a deficiency in beta protein production in hemoglobin results in severe anemia, making the individual reliant on regular blood transfusions and continuous medical care such as iron chelation therapy, surgical options like splenectomy and stem cell transplantation [1].

Thalassemia poses a significant health threat, affecting 72% of the world’s 229 countries. Approximately 240 million people worldwide are carriers of beta thalassemia, with around 60,000 new thalassemia major recorded annually. A significant portion of these cases, around 55 million, are found in the thalassemia belt, particularly in Southeast Asia [2].

Indonesia is among the countries in the ‘Thalassemia Belt,’ with a β-thalassemia (β-thal) prevalence ranging between 5.0% and 10.0%. It is estimated that there are 1,035 β-thal patients in the country, with approximately 43.0% registered in West Java [3]. According to the Indonesian Thalassemia Foundation, the number of thalassemia survivors reached 7,670 in 2015, with the majority located on Java Island (5,323 people) and a significant portion of them in West Java Province. By January 2016, West Java had 3,300 survivors, the highest number in Bandung City and Regency (670 people). Garut Regency ranked fourth, and Sumedang Regency eighth, in the top ten areas with the highest cases. The Indonesian government has now set a goal of achieving zero births of individuals with thalassemia major in Indonesia [4]. Medical records from the primary referral hospital and treatment centre for thalassemia survivors in West Java showed that 230 survivors visited the Hemato-Oncology clinic in January 2013, with 70% of them being adolescents aged 14–21 [5]. A 2018 survey revealed that in Sumedang Hospital, out of 165 patients, 48 (29.2%) were adolescents; in Dr. Slamet Hospital Garut, 48 (19.6%) of the 246 patients were adolescents; and in Majalaya Hospital, 26 (26.6%) of the 98 patients were teenagers.

Thalassemia major negatively impacts physical and psychosocial well-being of thalassemic children and adolescents due to both the illness itself and the demands of its treatment [6]. Adolescents living with thalassemia major encounter numerous physical and psychosocial difficulties [7]. Physical abnormalities such as growth delays, hepatosplenomegaly, yellowish skin tone, and distinctive facial features marked by pronounced lines are common [6]. In addition to physical problems, they face psychological and social issues [8]. Physical appearance makes the physical changes, caused by blood transfusions potentially harmful to their body image or self-image. This altered body image often reduces individuals’ self-esteem and self-confidence, making them feel different from others. This situation can result in emotional conflict ant lead to psychological issues [9].

Psychological issues in thalassemia major survivors arise due to their heightened vulnerability to various stressors, including anxiety and fear [10]. Research has shown that these survivors often experience somatic complaints, low self-esteem, and anxiety related to their illness and its potential future implications [1, 9, 11–13]. If left unaddressed, anxiety can escalate to panic levels. Individuals with panic disorder may become unable to perform tasks even with proper guidance, ultimately leading to a diminished quality of life (QoL) [14].

A qualitative study involving interviews with adolescents diagnosed with thalassemia major highlighted their concerns about the potential consequences of the condition and the need for frequent blood transfusions. These adolescents voiced fears such as dying at a young age, feeling ashamed due to physical changes that distinguish them from their healthy peers, lacking self-confidence in social situations, and often feeling weak. During the interviews, most adolescents avoided eye contact and gave minimal responses. Nurses need to understand the challenges these individuals face, while parents and families should offer support, collaborate during therapy, and engage in discussions about the emotional impact of the disease, its treatment, and its effects on their lives [15]. Therefore, nurses play a vital role in assessing the severity and frequency of anxiety experienced by survivors, identifying the underlying factors, exploring their coping strategies, and evaluating the overall impact of this anxiety [13, 14, 16].

Somatic anxiety factors can be evaluated through psychological anxiety factors, such as body image, self-image, and self-esteem [17]. The psychological aspect of anxiety poses a threat to self-esteem, making individual coping strategies and social support crucial. Courageous coping served as a partial mediator in the connection between social support and resilience [18]. Courageous coping enhances resilience, while defensive coping contributes to reduced resilience [7].

The researcher examined these four factors in relation to anxiety levels. These elements distinguish this study from previous research. While studies on anxiety in thalassemia major survivors have primarily been conducted abroad, research on factors contributing to anxiety in adolescents with thalassemia major is still relatively rare in Indonesia. Understanding the factors related to anxiety in adolescents and identifying the most dominant ones is essential for developing interventions to prevent anxiety and panic. This study, therefore, aimed to investigate the factors affecting anxiety levels in adolescent survivors of thalassemia major.

## Materials and methods

### Study design

This study employed a quantitative method with a correlational analytical design using a cross-sectional approach [19–21]. The variables examined included independent variables such as body image, self-esteem, coping strategies, and social support, as well as the dependent variable, which was the level of anxiety among adolescent thalassemia major survivors.

### Participants and setting

This study’s population consisted of all adolescent thalassemia survivors who were documented in medical records and regularly received blood transfusions at several referral hospitals in Indonesia, including Sumedang Hospital, Majalaya Hospital, Dr.Slamet Garut Hospital, and Guntur Hospital. The sampling process was conducted over a two-month period, from June to July 2018. This study’s minimum required sample size was 91 participants, leading to total sampling as the sampling technique.

The sample size for this study was determined through power analysis, a robust method for calculating sample size in research involving multiple regression analysis. Power analysis estimates the sample size required to reject the null hypothesis that the R value equals zero by considering the effect size, the number of predictors, the desired power, and the significance level (α). In multiple regression, the effect size is expressed as the R² value, which can be derived from previous studies or based on conventional standards: small effect size (R² = 0.02), medium effect size (R² = 0.13), or large effect size (R² = 0.30) (Polit & Beck, 2012). For this study, the parameters included a significance level (α) of 0.05, desired power (1-β) of 0.80, and a medium effect size (R² = 0.13), as commonly used in nursing research (Polit & Beck, 2012). Using these values (R² = 0.13, α = 0.05, β = 0.02, Zα = 1.96, Zβ = 0.84, I = 4), the minimum sample size was calculated to be 91 respondents. This calculation was derived by aligning the specified parameters with sample size estimation tables for 2 to 10 predictors and various R² values at a power of 0.80 and an alpha level of 0.05 (Polit & Beck, 2012). Cohen et al. (2007) emphasize that larger sample sizes relative to the population yield more reliable results. Consequently, this study employed a total sampling method, including all eligible adolescents with thalassemia major (aged 13–20 years) from RSUD Sumedang, RSUD Majalaya, RSUD dr. Slamet Garut, and RS Guntur Garut, resulting in a total of 122 respondents. Therefore, the final sample included all adolescent survivors of thalassemia major, aged 13 to 20 years, totaling 122 individuals.

### Instrument

The research utilized the Hamilton Anxiety Scale, Body Image Scale, Rosenberg Self-Esteem Scale, Ways of Coping Questionnaire, and the Multidimensional Scale of Perceived Social Support for measurements. The content validity of the instrument was assessed by supervising lecturers to ensure the items were appropriate for measuring the intended variables. Construct validity was empirically tested on 35 adolescents with thalassemia major at RSUD Gunung Jati, Cirebon. This test employed the Pearson Product Moment method (Hastono, 2013), where each item within a variable was correlated with its total score. The resulting correlation coefficients were compared to the r-table value to determine item validity. With a significance level of α = 0.05 and degrees of freedom (n–2) for a sample size of 35, the r-table value was set at 0.283. Items were considered valid if the calculated r-value exceeded the r-table value. Specifically, the Hamilton Anxiety Questionnaire (14 items), the Body Image Scale Questionnaire (10 items), the Rosenberg Self-Esteem Scale (10 items), the Ways of Coping Questionnaire (66 items), and the MSPSS Questionnaire (12 items) all demonstrated item validity. Reliability testing, conducted using Cronbach’s Alpha, showed values greater than 0.7 for all instruments, indicating that the items measuring anxiety, body image, coping strategies, self-esteem, and social support were reliable and appropriate for the study. These instruments were valid and reliable.

Instrument reliability was assessed using Cronbach’s Alpha. According to Santosa & Hidayat (2014), an instrument is deemed reliable if the Cronbach’s Alpha value is ≥ 0.7; values below 0.7 indicate unreliability. The analysis revealed that the Cronbach’s Alpha values for all instruments exceeded 0.7, confirming the reliability of the items measuring the five variables—anxiety, body image, coping, self-esteem, and social support—making them appropriate for use in the study.

The completion process for the questionnaires involved the researcher providing clear instructions to respondents. Participants were required to complete questionnaires covering topics such as thalassemia major, anxiety (using the Hamilton Anxiety Questionnaire), body image (Body Image Scale), self-esteem (Rosenberg Self-Esteem Scale), coping strategies (Ways of Coping Questionnaire), and perceived social support (Multidimensional Scale of Perceived Social Support). Respondents were allocated 60 min for this task. During the process, the researcher remained available to help as needed, and a nurse from the thalassemia clinic, who had been briefed on the study procedures, also supported respondents. Afterward, the researcher thoroughly reviewed the completed questionnaires to ensure they were filled out completely and clearly. Responses were then checked for consistency before being accepted into the study’s dataset.

### Ethic approval and consent to participate

The Research Ethics Committee at Universitas Padjadjaran granted ethical approval for this study under reference number 570/UN6.KEP/EC/2018. This research adhered to the Five Rights of Human Subjects in Research [22]. Written informed consent was obtained from all participants. For participants under 18, informed consent was obtained from their parents. All participants were informed about the study’s purpose and methods and were assured of their anonymity and the confidentiality of their data.

### Data analysis

The data were analyzed using several methods: univariate analysis through frequency distribution and percentage, bivariate analysis using the chi-square test, and multivariate analysis through logistic regression. The multivariate analysis was employed to examine the impact of the independent factors or variables on the dependent variable. In this research, the dependent variable, the anxiety level, was represented as categorical data, making logistic regression the appropriate multivariate analysis method. Logistic regression is conducted when more than one independent variable has a *p*-value of less than 0.250 in the bivariate test, qualifying it as a potential predictor. If fewer than two variables meet these criteria, logistic regression analysis cannot be performed.

## Results

### Characteristics of participants

More than half of the population, 54.10%, were aged 13–15 years (early adolescence). Additionally, 56.60% of the population were female. The most common level of education was Elementary School, representing 33.6% of the participants. The duration of diagnosis ranged from 1 to 20 years. Nearly all adolescents, 93.40%, did not experience complications (as shown in Table 1).

**Table 1:** Characteristics of adolescents with Thalassemia based on age, gender, Education, duration of diagnosis and Disease complications (*n* = 122)

Variable	Frequency (*n*)	Percentage (%)
**Age**		
13–15 years 16–18 years 19–20 years	66 40 16	54,1 32,8 13,1
**Gender**		
Male Female	53 69	43,4 56,6
**Last Education**		
Not Educated Elementary School Junior High School Senior High School Perguruan Tinggi	17 41 39 23 2	13,9 33,6 32,0 18,9 1,6
**Duration of Diagnose**		
Median (Range in Reays)	13 (1–20)	
**Disease Complication**		
Yes No	8 114	6,6 93,4

### The anxiety levels of adolescent thalassemia major survivors in West Java

Most adolescent thalassemia major survivors in West Java, 70.50%, experience mild anxiety, medium anxiety (9,80%) and severe anxiety (19,70%) (as shown in Table 2).

**Table 2:** The anxiety levels of adolescent Thalassemia Major survivors in West Java (*n* = 122)

Variable	Category	Frequency (%)
**Level of Anxiety**	Mild	86 (70,5)
	Medium	12 (9,8)
	Severe	24 (19,7)

### Analysis of the relationship between variables and anxiety levels among adolescent thalassemia major survivors in West Java

The bivariate analysis of the correlation test between variables and anxiety levels reveals a highly significant relationship between body image, self-esteem, and coping strategies with the anxiety levels of adolescent thalassemia major survivors in West Java, all with *p*-values < 0.05 (*p* < 0.001, *p* = 0.002, and *p* = 0.036, respectively). However, the correlation test for social support and anxiety levels yielded a *p*-value > 0.05, indicating no significant relationship between social support and anxiety levels among these adolescents (see Table 3).

**Table 3:** Correlation test of variables with anxiety levels of adolescent Thalassemia Major survivors in West Java (*n* = 122)

Variable	Frequency (*n*)	Level of Anxiety		*p*-value
		Mild (*n* = 86)	Medium-severe (*n* = 36)	
**Body Image**				
Good	105	82 (78,1)	23 (21,9)	< 0,001
Bad	17	4 (23,5)	13 (76,5)	
**Self Esteem**				
Low	26	12 (46,2)	14 (53,8)	0,002
High	96	74 (77,1)	22 (22,9)	
**Coping**				
Problem	75	58 (77,3)	17 (22,7)	0,036
Emotion	47	28 (59,6)	19 (40,4)	
**Social Support**				
Low/Medium	35	23 (65,7)	12 (34,3)	0,463
High	87	63 (72,4)	24 (27,6)	

### Factors that the most influential anxiety levels of adolescent thalassemia major survivors in West Java

The bivariate analysis results indicate that three independent variables have a *p*-value of less than 0.250: body image (< 0.001), self-esteem (0.002), and coping (0.036). This allows for logistic regression analysis to be performed with these three variables. Using the backwards method, the logistic regression analysis results show that only body image significantly affects anxiety levels, with a *p*-value of < 0.01 (< 0.001). In contrast, self-esteem and coping do not significantly influence anxiety levels. According to the adjusted Odds Ratio (OR), the body image variable has an OR of 11.6, suggesting that adolescent Thalassemia Major survivors with poor body image are 11.6 times more likely to experience severe anxiety levels (see Table 4).

**Table 4:** The logistic regression analysis of multivariate factors that the most influential on anxiety levels of adolescent Thalassemia Major survivors in West Java

	Independent Variable	Adjusted OR (95% CI)	*p*-value
**Early Model**	Body Image	8,9 (2,4–32,7)	0,001**
	Self Esteem	0,6 (0,2–1,9)	0,376
	Coping	1,9 (0,8–4,7)	0,161
**Last Model**	Body Image	11,6 (3,4–38,9)	< 0,001**

## Discussion

### Anxiety among adolescent thalassemia major survivors

The descriptive analysis results in this study revealed that all adolescents with thalassemia major experienced varying levels of anxiety, ranging from mild to severe. The majority, 70.50%, experienced mild anxiety, 9.80% had medium anxiety, while 19.70% faced severe anxiety. This anxiety arises as a response to the identity-threatening effects of Thalassemia Major and its treatment. The condition is marked by negative emotions, such as worry and fear, and is often accompanied by physical tension. The chronic nature of thalassemia, the lifelong need for regular transfusions, and associated complications negative impact on an adolescent thalassemia survivor’s physical health, psychological state, independence, social relationships, personal beliefs, and overall quality of life, including interactions with their environment. In line with their developmental stage, anxiety in these adolescents is strongly linked to concerns about rejection by peers. During early to middle adolescence, individuals are particularly focused on body changes, acceptance by peers, the struggle to master skills within peer groups, adjusting to changes in body image, and developing attraction to the opposite sex [6].

Severe anxiety levels are influenced by factors such as age, clinical conditions or disease complications, and non-compliance with blood transfusions and iron chelation therapy. In this study, 13.10% of late adolescents (aged 19–20) experienced complications like heart disease, and 6.60% faced other illnesses, which may contribute to their severe anxiety. This finding aligns with research from Iran, where the average anxiety score was higher in thalassemia patients aged 19 and older. The relationship between complications and anxiety is evident, as patients with complications tend to have higher anxiety scores [23]. Other study also show that with increasing age, the prevalence of diabetes and hepatitis rises, leading to heightened anxiety in older thalassemia survivors [24].

Thalassemia patients with severe negative symptoms probably feel unable to control stress and tend to ignore their problems instead of using positive coping methods. Defensive coping showed a positive and significant relationship with courageous coping. Whenever a person engages in emotional and avoidant coping, they try to manage these emotional situations with courageous coping strategies, however, in the future, if this tense situation continues, the use of courageous coping strategies in people decreases. These coping strategies play vital roles in helping to achieve resilience. Defensive coping can be converted to courageous coping if individuals are able to expand other protective factors to buffer the risk factors. Social support seemed to play a role in changing defensive coping to courageous coping [18].

Several factors contribute to this anxiety, frequent hospitalizations for transfusions, activity limitations, pain, side effects of iron chelation therapy such as Cooley’s facies, delayed puberty, complications such as heart failure, as well as physical attributes like bronze-coloured skin, short stature, and fear of death, age, gender, and coping mechanisms can contribute to feelings of anxiety [23–26]. Complications arising from thalassemia affect the physical, psychological, and social aspects of life, leading to emotional conflicts, including heightened anxiety [1].

### Factors associated with anxiety levels in adolescent thalassemia major survivors

The bivariate analysis results revealed a highly significant relationship between the variables of body image, self-esteem, and coping strategies with anxiety levels. Adolescent survivors of thalassemia may experience physical changes or scars due to repeated medical procedures, affecting their self-image and self-esteem. Concerns about appearance can be heightened during adolescence, a period when identity and body image are especially sensitive, 70.70% of individuals with thalassemia have low self-esteem, which is also linked to their high levels of anxiety [27]. Another study found that anxiety in thalassemia major patients was significantly associated with low self-esteem, identifying it as an independent predictor of anxiety [25]. In this research, most adolescents employed problem-focused coping strategies. This approach reduces stressors by learning new skills or methods to adapt to changing situations, circumstances, or challenges. Adolescents are more likely to use this strategy if they believe they can quickly alter the situation, as it focuses on completely resolving problems to alleviate anxiety [28]. A previous study demonstrated that teaching coping strategies effectively enhances the ability to manage stress and other complications in individuals with thalassemia major. Moreover, these strategies can increase problem-focused coping and reduce reliance on emotion-focused coping strategies [26].

This current study findings did not reveal a significant relationship between social support and the anxiety levels of adolescent thalassemia survivors in West Java. This may be due to the way individuals perceive support from family, friends, and significant others, as perceptions can vary significantly from person to person. Moreover, Anxiety is multifaceted and influenced by various factors such as biological predispositions, coping mechanisms, personality traits, or external stressors (e.g., financial stress, health concerns). These factors might overshadow the potential benefits of social support in reducing anxiety. This research evaluated social support based on contributions from family, friends, and special individuals. Social support, as an external factor, does not directly influence anxiety but can enhance self-esteem, body image, and the adoption of effective coping strategies [14]. Adolescents with thalassemia major require comprehensive support from family, friends, and significant others as a source of motivation, particularly for adherence to blood transfusions and iron chelation therapy [29]. Social support is crucial for individuals with thalassemia major, as they require lifelong psychological support, especially from their families, to improve adherence to therapy, well-being, and treatment outcomes [25].

### Key factors influencing anxiety levels in adolescent thalassemia major survivors

In this research, body image emerged as the most dominant factor associated with anxiety levels in adolescents with thalassemia major, showing a highly significant relationship. According to the OR, adolescents with a poor self-image are 11.6 times more likely to experience severe anxiety. This indicates that severe anxiety is more prevalent among those with a negative self-image. Their physical condition heavily influences their self-image. Since physical appearance plays a crucial role in adolescent development, changes in physical condition contribute to a poor body image, leading to emotional conflicts and psychological issues, including anxiety [8, 9, 25].

Adolescents with thalassemia major often face anxiety related to body image disturbance due to the visible and physical changes associated with the condition and its treatment. The negative impact of treatment, especially regular blood transfusions and iron chelation therapy can cause skin discoloration or scarring at injection sites, both of which can affect appearance. Adolescents may worry about appearing “sick” or “different,” impacting their self-esteem and confidence. Adolescence is a time when physical appearance is particularly important, and societal standards of beauty can pressure teens. The physical effects of thalassemia can lead to feelings of inadequacy and anxiety, as they feel they do not measure up to these standards. Adolescence is a critical period for developing self-identity. Physical changes due to thalassemia can make it harder for adolescents to feel comfortable with their bodies, affecting their self-image and how they define themselves. Knowing that these physical changes are lifelong, and may worsen over time, can intensify anxiety. This creates a cycle where worry over body image and health exacerbates anxiety, which can further impact their mental and emotional well-being of adolescent’s survivor thalassemia major.

The combination of these physical, social, and emotional challenges makes adolescents with thalassemia particularly vulnerable to anxiety related to body image. Supportive counseling, positive coping strategies, and a strong support system can play a vital role in helping them manage this anxiety and improve their self-image.

### Strengths and limitations of the study

This study has several limitations, including focusing on only four hospitals in West Java, Indonesia. However, it also has notable strengths. The research was conducted in both general and referral hospitals, allowing it to provide a broader picture of anxiety issues among adolescent thalassemia survivors in West Java and potentially across Indonesia. Additionally, these findings offer valuable insights that can support efforts to address the challenges faced by adolescent thalassemia survivors in Indonesia.”

### Implications of study

The current study has highlighted several factors that contribute to anxiety in adolescent Thalassemia survivors. These findings carry important implications for both healthcare providers and policymakers. For healthcare workers, understanding these factors can help tailor interventions and provide more personalized care that addresses the specific needs of adolescents struggling with anxiety due to thalassemia and its treatment. This might include developing targeted mental health support programs, enhancing communication with patients about their physical and psychological concerns, and integrating anxiety management strategies into routine care for thalassemia patients.

On the policy level, the study’s insights can guide the government in formulating comprehensive health policies that prioritize mental health support for thalassemia patients. This could involve allocating resources for specialized counselling services in hospitals, conducting awareness campaigns about the psychological impact of chronic diseases like thalassemia, and ensuring that adolescent thalassemia survivors have access to community-based support systems. Particularly in West Java, where this study was conducted, these strategies could be crucial in addressing the anxiety-related challenges faced by these adolescents. Ultimately, the findings of this study provide a critical evidence base that can help shape effective strategies and interventions aimed at reducing anxiety among adolescent thalassemia survivors, thereby improving their overall QoL.

## Conclusion

Adolescents with thalassemia major in West Java primarily experience mild anxiety, with the study showing strong links between anxiety levels and factors like body image and self-esteem. This suggests that how these adolescents perceive their physical appearance and self-worth is vital to their mental well-being. Coping strategies also significantly correlate with anxiety, highlighting the need for effective methods to manage stress and emotional challenges. In contrast, social support does not appear to significantly impact anxiety in this group. Body image emerges as the most influential factor, with poor body image notably increasing the likelihood of severe anxiety. These findings emphasize the importance of providing thorough psychological support that addresses body image issues, fosters self-esteem, and strengthens coping skills to enhance the mental health of adolescent survivors. Further research on a national level, involving diverse districts or cities in Indonesia, is recommended to gain broader insights into this issue.

## Data Availability

Data in this study is provided within the manuscript.

## Abbreviations

HbF: Fetal hemoglobin
HbA: Hemoglobin A
β-thal: β-thalassemia
QoL: Quality of life
OR: Odds Ratio

## References

[1] Shahraki-vahed A, Firouzkouhi M, Abdollahimohammad A, Ghalgaie J. Lived experiences of Iranian parents of beta-thalassemia children. J Multidiscip Healthc. 2017;10:243–51. 28721064 10.2147/JMDH.S132848 PMC5499950

[2] Rafii Z, Ahmadi F, Nourbakhsh SMK. The effects of an Orientation Program on Quality of Life of patients with Thalassemia: a quasi-experimental study. J Caring Sci. 2016;5(3):223–9. 27752488 10.15171/jcs.2016.024 PMC5045956

[3] Panigoro R, Rakhmila LE, Sribudiani Y, Maskoen AM, Tjandraprawira KD. Thalassemia in Indonesia: Screening Program, diagnosis and research. Hemoglobin. 2019;43(6):305–305.

[4] Effendi S. Thalassemia Overview in West Java Province. In: Seminar Meet The Thalassemia Expert Optimizing Thalassemia Continuum Care. 2017.

[5] Maghfiroh R, Okatiranti, Sitorus RE, GAmbaran Harga Diri. Pasien thalasemia remaja (usia 14–21 tahun) di klinik hemato-onkologi rsup Dr. HASAN SADIKIN BANDUNG. Jurnal Ilmu Keperawatan. 2014;11(2).

[6] Kyle T, Carman S. Buku Ajar Keperawatan Pediatri. Jakarta: Penerbit Buku Kedokteran EGC; 2015.

[7] Parviniannasab AM, Rakhshan M, Momennasab M, Soltanian M, Bijani M. The relationship between coping strategies and resilience among adolescents with beta-thalassemia major. J Child Adolesc Psychiatr Nurs. 2021;34(4):329–34. 34137120 10.1111/jcap.12339

[8] Behdani F, Badiee Z, Hebrani P, Moharreri F, Badiee AH, Hajivosugh N et al. Psychological aspects in children and adolescents with major thalassemia: a case-control study. Iran J Pediatr. 2015;25(2). 10.5812/ijp.25(3)2015.322 PMC4505986 26199704

[9] Surilena, Peranan relasi keluarga pada psikopatologi remaja penderita talasemia. Damianus J Med. 2014;13(2):137–47.

[10] Kaheni S, Yaghobian M, Sharefzadah GH, Vahidi A, Ghorbani H, Abderahemi A. Quality of life in children with β-thalassemia major at center for special diseases. Iran J Pediatr Hematol Oncol. 2013;3(3):108–13. PMC3921875 24575281

[11] Yengil E, Acipayam C, Kokacya MH, Kurhan F, Oktay G, Ozer C. Anxiety, depression and quality of life in patients with beta thalassemia major and their caregivers. Int J Clin Exp Med. 2014;7(8):2165–72. 25232402 PMC4161562

[12] Hamilton M. The assessment of anxiety states by rating. Br J Med Psychol. 1959;32(1):50–5. 13638508 10.1111/j.2044-8341.1959.tb00467.x

[13] Mulyana AM, Rakhmawati W, Adistie F, Maulana S. Recent evidence regarding family-centred empowerment in improving Quality of Life and Treatment outcomes among Asian and African Children with Chronic diseases: a scoping review. J Heal Transl Med. 2024;27(1):7–20.

[14] Stuart G. Principles and practice of Psychiatric nursing. Edisi Indo. Mosby Elsevier; 2013.

[15] Mediani H, Nurhidayah I, Mardhiyah A, Panigoro R. An exploratory qualitative study on Indonesian mothers’ needs and concerns about having a thalassemic child and its treatment. Highlights on Medicine and Medical Science. Book Publisher International (a part of Sciencedomain International); 2021. pp. 45–56.

[16] Mulyana AM, Juniarti N, Rakhmawati W, Wartakusumah R, Fitri SYR. The efficacy of internet-based interventions in family-centered empowerment among children with chronic diseases: a mixed-methods systematic review. J Multidiscip Healthc. 2023;16:3415–33. 37964797 10.2147/JMDH.S440082 PMC10642539

[17] Zartaloudi A, Christopoulos D, Kelesi M, Govina O, Mantzorou M, Adamakidou T, et al. Body image, Social Physique anxiety levels and self-esteem among adults participating in Physical Activity Programs. Diseases. 2023;11(2):1–17. 10.3390/diseases11020066 PMC10204469 37218879

[18] Rambod M, Hamidizadeh S, Bazrafshan MR, Parviniannasab AM. Risk and protective factors for resilience among adolescents and young adults with beta-thalassemia major. BMC Psychol. 2023;11(1):231. 37568184 10.1186/s40359-023-01268-2 PMC10422764

[19] Seeram E. An overview of correlational research. Radiol Technol. 2019;91(2):176–9. 31685592

[20] Lau F. Handbook of eHealth evaluation: an evidence-based Approach. Francis Lau and Craig Kuziemsky. editor. University of Victoria, Columbia, Canada. Canada; 2016. p. 504. 29431951

[21] Devi B, Lepcha N, Devi R, Pradhan S, Giri D, Basnet S. Application of Correlational Research Design in nursing and application of Correlational Research Design in nursing. J Xi’an Shiyou Univ. 2023;65(11):60–9.

[22] Polit D, Beck C. Essentials of nursing research: appraising evidence for nursing practice. 7th ed. Wolters Kluwer Lippincot Williams & Wilkins; 2010.

[23] Adib-Hajbaghery M, Ahmadi M. Health Related Quality of Life, Depression, anxiety and stress in patients with Beta-Thalassemia Major. Iran J Pediatr Hematol Oncol. 2015;5(4):193–205. PMC4779154 26985352

[24] Seyedifar M, Dorkoosh FA, Hamidieh AA, Naderi M, Karami H, Karimi M, et al. Health-Related Quality of Life and Health Utility Values in Beta thalassemia major patients receiving different types of Iron Chelators in Iran. Int J Hematol stem cell Res. 2016;10(4):224–31. PMC5139942 27928477

[25] Yahia S, El-Hadidy MA, El-Gilany AH, Anwar R, Darwish A, Mansour AK. Predictors of anxiety and depression in Egyptian thalassemic patients: a single center study. Int J Hematol. 2013;97(5):604–9. 23595950 10.1007/s12185-013-1322-z

[26] Hashemi F, Naderi Darshori A, Sharif F, Karimi M, Zare N. Effect of coping strategies training on its use by Thalassemia major adolescents: a randomized controlled clinical trial. Int J Community Based Nurs Midwifery. 2015;3(1):67–74. 25553336 PMC4280559

[27] Yildiz D, Suluhan D, Fidanci BE, Kiziler E, Boyraz G. 6.16 determining the relationship between self-esteem, body image, and anxiety levels of children with thalassemia. J Am Acad Child Adolesc Psychiatry. 2016;55(10):S209.

[28] Lazarus RS, Folkman S. Transactional theory and research on emotions and coping. Eur J Pers. 1987;1(3):141–69.

[29] Pouraboli B, Abedi HA, Abbaszadeh A, Kazemi M. Living in a misty marsh: a qualitative study on the experiences of self-care suffering of patients with thalassemia. Iran J Nurs Midwifery Res. 2014;19(7 Suppl 1):S77–82. 25949257 PMC4402994

